# Proxy markers of serum retinol concentration, used alone and in combination, to assess population vitamin A status in Kenyan children: a cross-sectional study

**DOI:** 10.1186/s12916-014-0256-5

**Published:** 2015-02-11

**Authors:** Elise F Talsma, Hans Verhoef, Inge D Brouwer, Anne S Mburu-de Wagt, Paul JM Hulshof, Alida Melse-Boonstra

**Affiliations:** Division of Human Nutrition, Wageningen University, P.O. Box 8129, 6700 EV Wageningen, the Netherlands; Cell Biology and Immunology Group, Wageningen University, Wageningen, The Netherlands; Medical Research Council (MRC) International Nutrition Group, London School of Hygiene & Tropical Medicine, London, UK; Medical Research Council (MRC), Keneba, The Gambia

**Keywords:** Vitamin A deficiency, Discriminant analysis, ROC curve, Sensitivity and specificity, Predictive value of tests, Retinol, Retinol binding protein, Transthyretin, Inflammation, Diagnosis

## Abstract

**Background:**

Serum retinol concentration determined by high-performance liquid chromatography (HPLC) is recommended by the World Health Organization to assess population vitamin A status. This assay is expensive, technically demanding and rarely available in developing countries. Our objective was a) to assess the diagnostic performance of proxy markers in detecting vitamin A deficiency and b) to derive decision rules based on these markers to estimate vitamin A deficiency prevalence.

**Methods:**

A survey was conducted in 15 rural primary schools in Eastern Province, Kenya, with 375 children aged 6 to 12 years (25 randomly selected per school). Serum retinol concentration <0.70 μmol/L by HPLC was used to define vitamin A deficiency. Proxy markers for vitamin A deficiency were serum concentrations of retinol binding protein (RBP), transthyretin, retinol measured by fluorometry and RBP:transthyretin molar ratio.

**Results:**

The prevalence of vitamin A deficiency (HPLC) was 18%. Transthyretin and RBP showed the best diagnostic performance individually, with area-under-the-curve (AUC) values of 0.96 and 0.93. When combined, and with C-reactive protein added, the AUC increased to 0.98. A simple decision rule {(−15.277 × [RBP, μmol/L] - 7.013 × [Transthyretin, μmol/L] + 0.367 × [C-reactive protein, mg/L] + 24.714) > 0.496} yielded prevalence estimates of vitamin A deficiency that is unbiased by diagnostic error.

**Conclusions:**

The combination of transthyretin, RBP and C-reactive protein concentrations could eventually replace retinol concentration by HPLC in resource-poor settings as the preferred method to assess the population burden of vitamin A deficiency.

**Electronic supplementary material:**

The online version of this article (doi:10.1186/s12916-014-0256-5) contains supplementary material, which is available to authorized users.

## Background

A serum retinol (vitamin A) concentration of ≤0.70 μmol/L is recommended by the World Health Organization as a marker to assess the population burden of vitamin A deficiency [1,2]. Its measurement requires high-performance liquid chromatography (HPLC), which is expensive, technically demanding and rarely available in developing countries [3]. In addition, measurement of serum retinol concentration typically requires venipuncture to obtain the blood volume (>500 μL) necessary, and samples must be stored in tubes impermeable to light until laboratory analysis.

Several serum indicators proposed as proxy markers of vitamin A status may be conveniently used in resource-poor settings. These include retinol binding protein (RBP) concentration, the molar ratio of RBP:transthyretin and retinol concentration measured by fluorometry [4]. RBP is a transporter protein that binds, transports and delivers retinol to target organs. Its secretion from the liver, where it is produced, into the circulation depends on circulating retinol levels [5]. Studies in rats suggest that RBP is present in serum in a 1:1 molar ratio to retinol, but surveys in humans indicate that this ratio can be different and is influenced by inflammation, protein-energy malnutrition, obesity, vitamin A status, iron status and pregnancy [6]. Thus the molar concentration of retinol in serum can differ from that of RBP.

Transthyretin is involved in transport of retinol through the formation of a complex with RBP and retinol, which prevents the glomerular filtration of the RBP molecule in the kidneys [7,8]. The molar ratio of RBP:transthyretin has been proposed as an indicator of vitamin A status unaffected by inflammation [9]. Both RBP and transthyretin can be measured relatively easily by enzyme-linked immunosorbent assay (ELISA). Fluorometry exploits the characteristic of retinol to fluoresce under the influence of ultraviolet light, particularly when bound to RBP [10,11], allowing its measurement using a point-of-care test under field conditions.

To our knowledge, no studies have evaluated the diagnostic performance of combinations of these proxy markers to assess vitamin A status, and few studies have considered the effect of diagnostic error on prevalence estimates of vitamin A deficiency. The present study, conducted among Kenyan children, aimed to assess the diagnostic performance of the proxy markers listed above, alone or in combination, in detecting vitamin A deficiency defined as serum retinol concentration <0.70 μmol/L (measured by HPLC) [2]. In this analysis, we considered inflammation markers, age, body mass index for age z-score and iron status as additional diagnostic markers. Secondly, we aimed to derive decision rules based on these markers to estimate the prevalence of vitamin A deficiency.

## Methods

### Subjects and sample collection

The study was approved by ethical committees in Kenya and the Netherlands. We conducted a survey (June 2010) at 15 primary schools in Kibwezi and Makindu Districts in Eastern Province, Kenya, which had been selected from 45 public schools based on size (>350 children aged 6 to 12 years) and having no school feeding program. For each school, we randomly selected 25 children from an enrolment list of all children aged 6 to 12 years (n = 375), and we included those who were apparently healthy and without fever (ear drum temperature <37.5°C) upon examination by the research physician, and whose guardians had provided prior informed consent. Venous blood (6 mL) was obtained from each fasted child and kept shielded from light at 2 to 8°C for 30 to 60 min. After centrifugation (1200 *g*, 10 min), the serum was kept for 4 to 8 h at 2 to 8°C and subsequently stored in liquid nitrogen (−196°C) in Kenya, and at −80°C during transport and storage in the Netherlands. Blood samples were obtained by finger prick to measure haemoglobin concentration (HemoCue, Ängelholm, Sweden). Weight and height were measured according to WHO guidelines [12] to the nearest 0.1 kg and 0.1 cm using a mechanical floor scale and a portable stadiometer (Seca, Hamburg, Germany).

### Biochemical analyses

Concentrations of retinol (by HPLC), RBP and ferritin were determined at Wageningen University, the Netherlands (August 2010). Samples used to measure retinol concentrations were processed under subdued yellow light.

We added 200 μL sodium chloride (0.9% w/v in water) and 400 μL 96% ethanol, containing retinyl acetate as an internal standard, to 200 μL serum. Serum samples were extracted twice with 800 μL hexane for 5 min using a horizontal laboratory shaker (Edmund Buehler, model SM25, Heckingen, Germany) at 250 reciprocations/min, and then centrifuged for 2 min at 3000 *g*. The hexane supernatants were pooled into an HPLC vial. Twenty-five μL of the extract was injected directly into a polar BDS Hypersil CN HPLC column (150 × 3 mm inner diameter, particle size 5 μm) with a Javelin NH2 guard column (both from Keystone Scientific, Bellefonte PA, USA). The HPLC system (Spectra, Thermo Separation Products Inc., San Jose CA, USA) was equipped with two pumps (model P2000), a solvent degasser (model SCM400), a temperature-controlled auto sampler (model AS3000), a UV-visible forward optical scanning detector (UV3000), interface (model SN4000) and control and integration software (Chromquest 5.0). As eluent, we used a mixture of hexane-isopropanol (98.5%:1.5% v/v) containing triethylamine (0.1% v/v) as a mobile phase additive to reduce peak tailing, at a constant flow of 0.7 mL/min. Separations were measured at 325 nm and quantified using the internal standard method against retinol standards. The total runtime was 5 min. Within-run and between-run coefficient of variations (CV) were 1.6% and 2.1%, respectively, based on in-house control serum. Analysis of standard reference material SRM 968e from the National Institute of Standards and Technology (NIST, Gaithersburg, MD, USA) revealed deviations of 0.3%, 0.2% and 5% from certified values for the low, medium and high levels (1.19 μmol/L, 1.68 μmol/L and 2.26 μmol/L, respectively). Duplicate measurements were done on 10% of samples, resulting in a mean CV of 2.0%.

RBP concentrations were determined by immunoassay (catalogue DRB400, Quantikine, R&D Systems, Minneapolis, USA). Results were read in duplicate for 10% of samples. The inter-plate CV for six plates was 10.4%. The intra-assay CV for duplicate samples was 6.0%.

Ferritin concentrations were determined by enzymatic immunoassay (Ramco Laboratories, Stafford, TX, USA). Results were read in duplicate for 10% of samples. The inter-plate CV for six plates was 8.8%. The intra-assay CV for duplicate samples was 9.7%.

A point-of-care fluorometer (iCheck™ FLUORO; BioAnalyt, Teltow, Germany) was validated (see online Additional file 1) and used (September 2011) to measure concentrations of vitamin A (retinol and retinyl palmitate) at excitation and emission wavelengths of 330 nm and 470 nm. Children were ranked on serum retinol concentration and a subset of 105 samples was selected by taking every third sample. If the sample was insufficient, the next sample on the list was taken to ensure the same concentration range. 250 μL of serum was injected into a sealed glass cuvette prefilled with a proprietary reagent (IEX™ MILA, BioAnalyt) comprising a mixture of alcohols and organic solvents. 250 μL of phosphate buffered saline solution (PBS) was added to obtain the required 500-μL sample volume and the result was multiplied by two. Samples were measured according to manufacturer guidelines. Control samples provided by the manufacturer were measured at the beginning and end of each batch of measurements and were within the expected range.

Serum concentrations of transthyretin, C-reactive protein and α_1_-acid glycoprotein were determined by immunoturbidimetric assays on a Cobas Integra 800 system (Roche Diagnostics, Mannheim, Germany) at University Medical Centre, Leiden, The Netherlands (October 2010). The transthyretin concentration was measured using the PREA assay (Roche), with CVs of 1.9% and 3.2% at concentrations of 4.7 μmol/L and 11.4 μmol/L. The C-reactive protein concentration was measured by Tina-quant ultrasensitive assay (Roche), with CVs of 1.8% and 1.9% at concentrations of 3.98 mg/L and 12.81 mg/L. The α_1_-acid glycoprotein concentration was measured using the Tina-quant AAGP2 assay (Roche), with CVs of 1.3% and 0.5% at concentrations of 0.77 g/L and 1.27 g/L.

### Statistical analyses

Anthropometric z-scores were calculated using Anthro-plus (WHO, version 3.2.2). The results were analysed using the statistical software packages IBM SPSS 20.0 and STATA 12. Comparisons were done separately for all children and for those without inflammation, defined as serum concentrations of C-reactive protein <5 mg/L or α_1_-acid glycoprotein <1 g/L [13]. Distributions of serum markers were inspected by visual examination of histograms, and were described using conventional methods. We defined vitamin A status by serum retinol concentration (HPLC) <0.70 μmol/L (deficient) or ≥ 0.70 μmol/L (replete) [2]. Scatter plots and linear regression analysis were used to assess linearity in associations of the proxy markers with serum retinol concentration. Receiver operating characteristic (ROC) curves were used to assess the diagnostic accuracy of proxy serum markers in detecting vitamin A deficiency, whether alone or in linear combinations in comparison with retinol by HPLC. Diagnostic accuracy was determined by visual inspection of these curves and by assessing differences in the area under the curve (AUC) with corresponding *P*-values. A Bland-Altman plot was used to assess the agreement between measuring retinol concentration by HPLC and fluorescence [14].

Combinations of proxy markers may have better ability than single markers to distinguish between children with and without vitamin A deficiency. For pairs of markers, we assessed this distinguishing ability by visual inspection of scatter plots, with individuals being classified by vitamin A status. Logistic regression was used to assess the added diagnostic value of each marker and to produce linear predictors (combinations of diagnostic test results), which can be interpreted as decision rules to classify vitamin A status. Each newly defined linear predictor was used to compute the probability of vitamin A deficiency for all subjects, which can be considered on its own as the quantitative outcome of a new, stand-alone diagnostic test. Thus, we produced ROC curves by allowing this probability to vary within the range [0, 1]*.* Using a stepped-forward selection procedure, we started the model with the best proxy marker when used alone, and successively added other proxy markers, serum markers of inflammation, age, body mass index-for-age z-score and iron status as explanatory variables. We settled on a parsimonious model that only included markers found to have independent diagnostic value when used in combination with others, as judged by *P*-values for logistic regression coefficients.

We used two methods to assess the diagnostic accuracy of this parsimonious model. First, we assessed its goodness of fit by assessing the level of agreement between the probability of vitamin A deficiency as estimated by the model versus the actually observed frequencies. Thus, we ordered individuals and grouped them into deciles based on the predicted probability of vitamin A deficiency as derived from the logistic regression model, and plotted the mean predicted value in each decile against the frequency of vitamin A deficient cases that was actually observed in each decile. The resulting plot should ideally have a slope of 1 and an intersect of 0.

Second, we assessed the ability of the model to discriminate between children with or without vitamin A deficiency by means of an ROC plot and its AUC. With this model, we calibrated the value of the linear predictor to produce prevalence estimates of vitamin A deficiency that are unbiased by diagnostic error.

**Figure 1:**
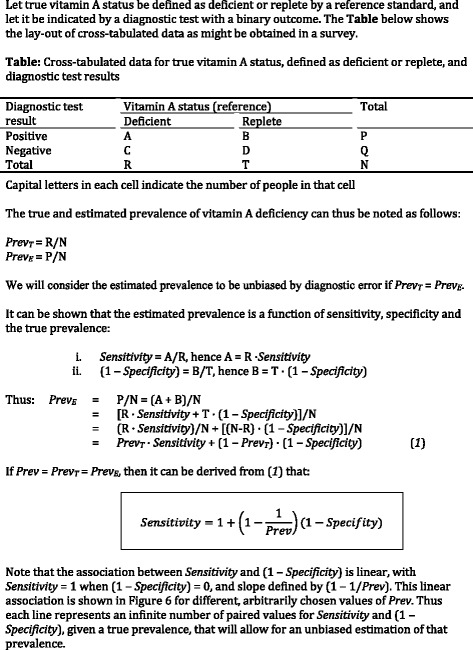
**Elimination of diagnostic error when estimating the prevalence of vitamin A deficiency.**

Given a diagnostic test with a binary outcome, a set of paired values for sensitivity and specificity exists that leads to a prevalence estimate that is identical to the true prevalence (Figure 1). The intersection of this set and the ROC curve obtained with our parsimonious logistic regression model indicates the value of the linear predictor (and thus the diagnostic decision rule) that would result in a prevalence estimate of vitamin A deficiency that is unbiased by diagnostic error. We calibrated the linear predictor to estimate the prevalence of vitamin A deficiency, with true prevalence arbitrarily selected as 6% and 15%, the mid-points for the ranges that indicate mild and moderate public health problems (2 to 10% and 10 to 20%, respectively) [2]. Similarly, we used 30% and 40% as an arbitrarily selected prevalence in the range (>20%) indicating a severe public health problem.

## Results

Complete data were collected for 372 children; for three children, no blood sample could be obtained. The frequency distribution of serum retinol at baseline is shown in online Additional file 1. Table 1 shows the characteristics of the total study population and the subsample (n = 105) for which retinol concentration was measured by fluorometry. Vitamin A deficiency occurred in 18% of children. Only 10% had inflammation; their exclusion from analysis led to similar estimates for concentrations of retinol (whether HPLC or fluorometry), RBP, transthyretin, RBP:transthyretin molar ratio and prevalence of vitamin A deficiency.

**Table 1:** **Characteristics of the study population**

**Main study**	**All children**	**Children without inflammation** ^**1**^
n	372	336
Age, years	9.2 (1.9)	9.2 (1.9)
Sex, girls:boys	199:173 (54%:46%)	180:146 (54%:46%)
Serum retinol concentration by HPLC, μmol/L	0.87 (0.19)	0.88 (0.18)
Vitamin A deficiency^1^	68 [18%]	49 [15%]
Serum RBP concentration, μmol/L	0.67 (0.17)	0.68 (0.17)
Serum transthyretin concentration, μmol/L	3.0 (0.62)	3.0 (0.60)
RBP:transthyretin molar ratio	0.23 (0.04)	0.23 (0.04)
Serum C-reactive protein concentration >5 mg/L	17 [5%]	0 [0%]
Serum α_1_-acid glycoprotein concentration >1 g/L	33 [9%]	0 [0%]
Inflammation ^2^	36 [10%]	0 [0%]
Haemoglobin concentration, g/L	130 (11)	129 (12)
Serum ferritin concentration, μg/L (median, IQR)	19.8 (12.8, 30.2)	19.2 (12.0, 29.5)
BMI for age z-score	−1.29 (0.91)	−1.29 (0.89)
**Sub-study**		
n	105	94
Age, years	8.9 (1.9)	8.9 (1.9)
Sex, girls:boys,	64:41 (61:39)	58:36 (62:38)
Serum retinol concentration by HPLC, μmol/L	0.87 (0.18)	0.88 (0.17)
Serum retinol concentration by fluorescence, μmol/L	0.79 (0.30)	0.79 (0.30)
Serum C-reactive protein concentration >5 mg/L	4 [4%]	0 [0%]
Serum α_1_-acid glycoprotein concentration >1 g/L	10 [10%]	0 [0%]
Inflammation^2^	11 [11%]	0 [0%]
Haemoglobin concentration, g/L	130 (10)	130 (10)
Serum ferritin concentration, μg/L (median, IQR)	18.9 (11.4, 29.4)	18.2 (11.2, 27.7)
BMI for age mean z-score	−1.43 (0.91)	−1.43 (0.89)

Values indicate mean (SD) or n [%] unless indicated otherwise.

^1^Serum retinol concentration (HPLC) <0.70 μmol/L. ^2^Defined by serum concentrations of C-reactive protein >5 mg/L or α_1_-acid glycoprotein >1 μg/L.

**Figure 2:**
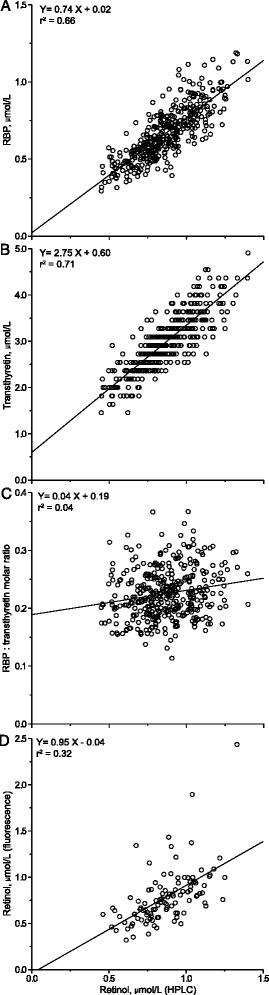
**Associations of four proxy markers with serum retinol concentration (HPLC). A**: RBP, **B**: transthyretin, **C**: RBP:transthyretin molar ratio, **D**: retinol by fluorescence.

In univariate analysis, retinol measured by HPLC was strongly associated with RBP and transthyretin, and to a lesser degree with retinol measured by fluorometry and the RBP:transthyretin molar ratio (Figure 2).

**Figure 3:**
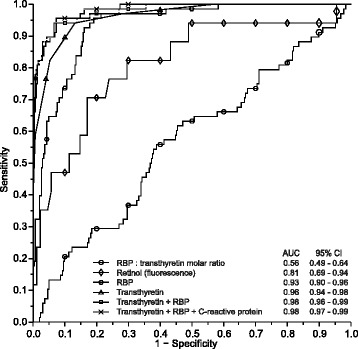
**ROC curves for proxy markers of vitamin A deficiency.**

Figure 3 shows the ROC plots for each proxy marker. The AUC was the highest for transthyretin and RBP (0.96 and 0.93, respectively), followed by retinol by fluorometry (0.81) and RBP:transthyretin molar ratio (0.56). Excluding children with inflammation resulted in a slight decrease in AUC for RBP and a slightly higher AUC for transthyretin and retinol by fluorometry, but did not appreciably change for the RBP:transthyretin molar ratio.

**Figure 4:**
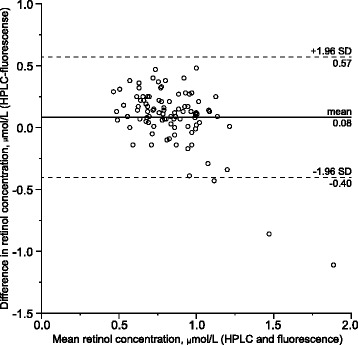
**Bland-Altman plot of retinol by HPLC versus fluorescence.** The difference between serum retinol concentrations obtained by HPLC and fluorometry (plotted on Y-axis) indicates by how much the former method is likely to differ from the latter method. The mean of these measurements (X-axis) is the best estimate of the true value, without assuming that one method is superior to the other. Assuming that the differences between results obtained with these methods are normally distributed, 95% of the differences will lie within the range that is indicated by the dotted lines.

The Bland-Altman plot (Figure 4) shows a mean difference of 0.083 μmol/L between the HPLC and fluorescence methods, with limits of agreement of −0.40 μmol/L and 0.57 μmol/L. The results of the two methods diverged with serum retinol concentration, indicating that the fluorescence method tended to overestimate concentrations.

**Figure 5:**
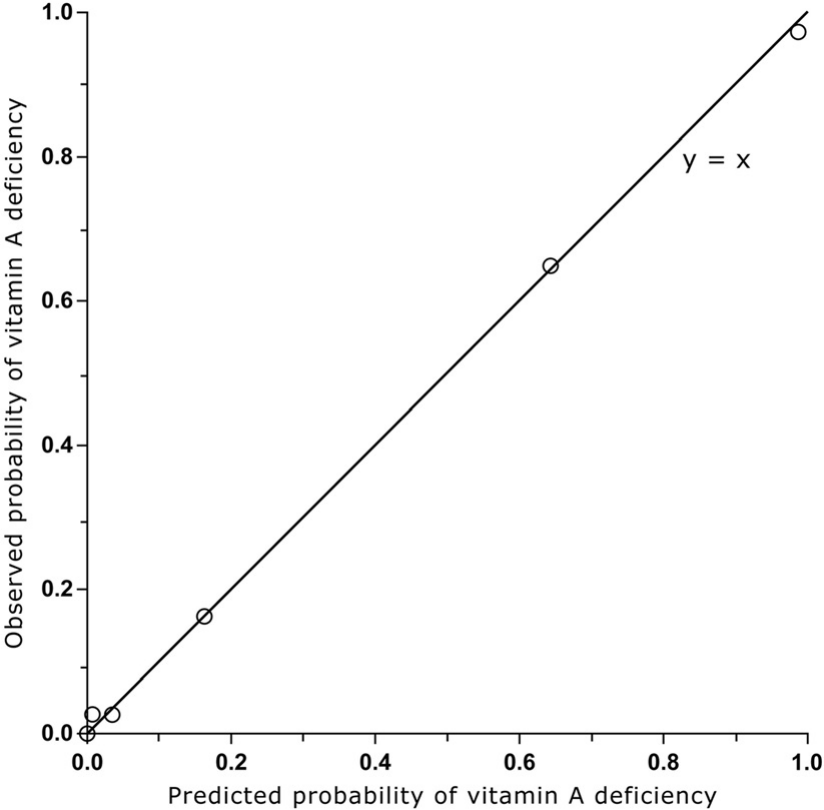
**Goodness of fit of the model in predicting vitamin A deficiency.** Predicted probability values are grouped in ten equal deciles based on increasing predicted probability values. Out of these ten groups, only six can be seen, as four groups contain the same predicted probability of 0 and overlap each other in this figure.

**Figure 6:**
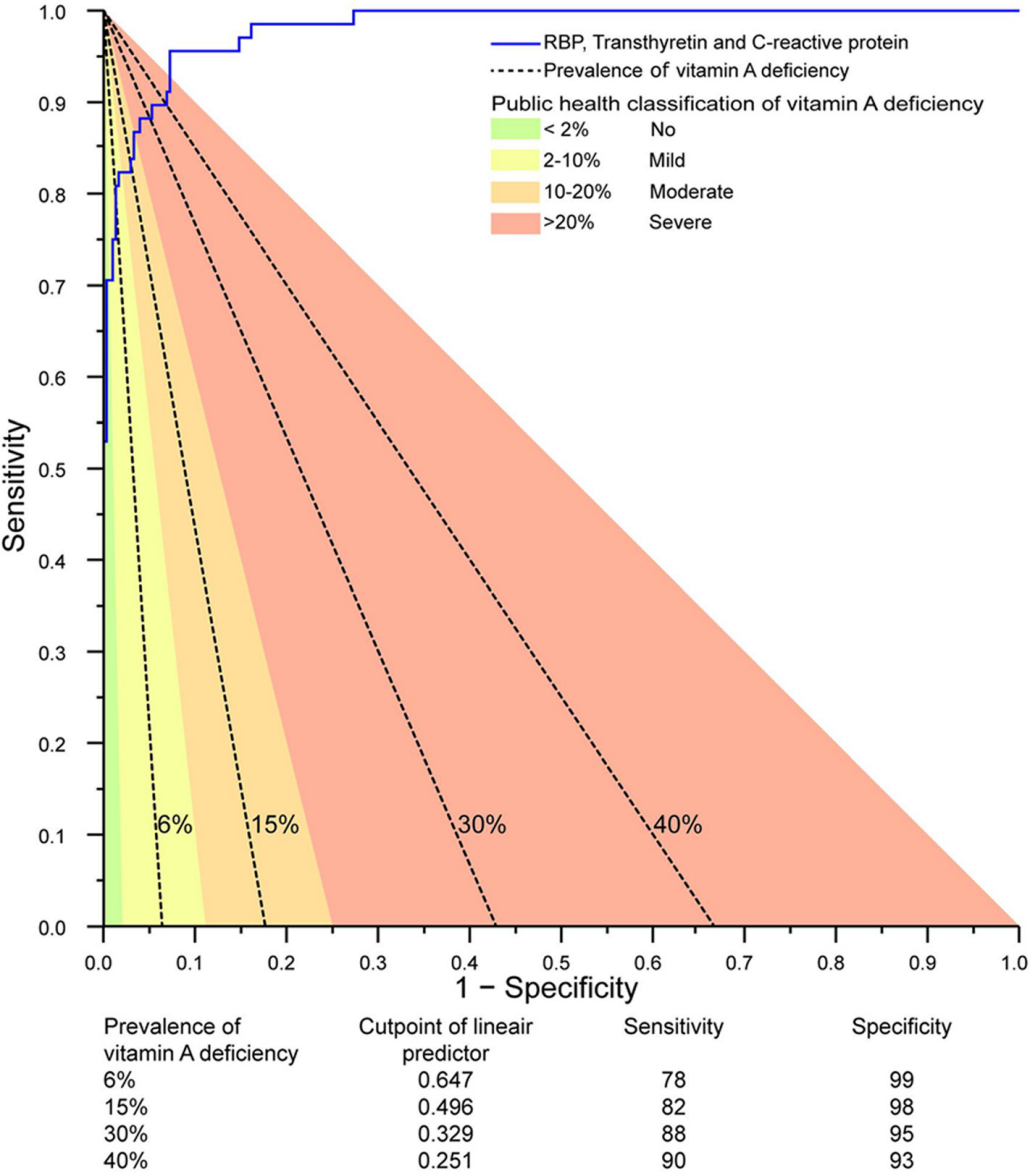
**ROC curve of the best model to predict vitamin A deficiency with its decision rules during different prevalence rates of vitamin A deficiency.** True prevalence was arbitrarily selected as 6%, 15%, 30% and 40% as the mid-points for the ranges that indicate mild, moderate and severe public health problems (2-10% and 10-20%, >20% respectively). Note that, with the true prevalence decreasing, the optimal cut-point for the linear predictor results in increased specificity even at the expense of sensitivity. For example, at a true prevalence of vitamin A deficiency of 15%, individuals for whom (−15.277 × [RBP_μmol/L_] - 7.013 × [Transthyretin_μmol/L_] + 0.367 × [C-reactive protein_mg/L_] + 24.714) > 0.496 should be classified as deficient, whereas all others can be classified as replete (in this formula, concentrations are indicated in straight brackets, and expressed in units as indicated).

Logistic regression resulted in a model of vitamin A deficiency dependent on RBP, transthyretin and C-reactive protein and a linear predictor of (−15.277 × [RBP_μmol/L_] - 7.013 × [Transthyretin_μmol/L_] + 0.367 × [C-reactive protein_mg/L_] + 24.714). Figure 5 illustrates that the observed versus the predicted probability of vitamin A deficiency were close to the line of identity, showing an excellent fit of the model. When used in combination, RBP and transthyretin were better at discriminating between children with and without vitamin A deficiency than when transthyretin was used alone (AUC: 0.98 versus 0.96; *P* = 0.01) or when RBP was used alone (AUC: 0.98 versus 0.93; *P* = 0.001) (Figure 3). Addition of C-reactive protein into the RBP and transthyretin model resulted in a marginal improvement of AUC but did not improve the model (AUC: 0.982 versus 0.979; *P* = 0.44). Figure 6 shows the decision rules-derived prevalence estimates for vitamin A deficiency, at true prevalence values of 6%, 15%, 30% and 40%, and the corresponding sensitivity and specificity values.

## Discussion

Serum concentrations of transthyretin and RBP, when used alone, performed well in discriminating between children with and without vitamin A deficiency. The test performance was even better when these markers were used in combination, and addition of serum C-reactive protein concentration could lead to further improvement, albeit marginally. We have shown how these three markers can be combined to estimate the prevalence of vitamin A deficiency in population surveys, based on a simple decision rule to determine individual vitamin A status.

The strong points of our study are: a) the novel use of combinations of multiple markers to determine vitamin A status; b) the relatively large sample size; c) the fact that the study population concerned children for whom vitamin A status is to be determined (as opposed to children with signs or symptoms suggesting deficiency, which may lead to biased estimates of diagnostic performance); d) the fact that the study goes beyond an assessment of diagnostic accuracy as indicated by sensitivity and specificity but demonstrates the application of a diagnostic strategy using these markers for public health purposes (estimation of the prevalence of deficiency).

Although transthyretin has been used as a vitamin A marker in many studies, it has mostly been analysed as the RBP:transthyretin molar ratio. Only one study reported the diagnostic performance of transthyretin [15], but it was based on univariate analysis and used the relative dose–response test as a reference, which had been questioned earlier as a valid indicator of vitamin A status [16]. Our results suggest that two to three proxy markers (serum concentrations of RBP, transthyretin and C-reactive protein) could replace serum retinol concentration measured by HPLC, with the advantages that these markers can be conveniently measured at relatively low cost by separate or multiplex ELISAs, and require only a small blood volume collected by finger puncture. Although transthyretin seems stable at refrigerated or frozen conditions for up to several weeks [17], additional studies are required to assess its stability under field conditions. Although serum C-reactive protein concentration had limited diagnostic utility in this study, we note that it may be more important in populations with higher prevalence and levels of inflammation. Our results indicate that the RBP:transthyretin molar ratio is inferior and should not be used.

Serum retinol concentration measured by fluorometry is also inferior, but its diagnostic utility may need re-assessment if the technology can be improved. The greatest divergence between retinol concentrations as measured by HPLC and those measured by fluorescence were at high concentrations, which suggests that our fluorescence results were affected for some children by retinyl esters derived from food. Such esters are not detected by our HPLC method, but they can increase fluorescence readings. We cannot exclude the possibility that some parents ignored our request to bring children in a fasted state.

Selection of cut-points for dichotomised diagnostic tests should depend on diagnostic aims. Vitamin A deficiency is defined by serum retinol concentrations <0.70 μmol/L because individuals who meet this criterion are considered to be at increased risk of morbidity and mortality [2]. To avoid missing cases, it may be desirable for a diagnostic test to have a high sensitivity in detecting such individuals, even at the expense of specificity. Another approach can be to maximise accuracy, i.e., the probability that individuals with and without vitamin A deficiency are correctly classified, which is appropriate if a false negative is considered to be equally undesirable as a false positive. In the present paper, our diagnostic aim was to estimate the prevalence of vitamin A deficiency without bias due to diagnostic error. Selection of cut-points to maximise either sensitivity or accuracy will lead to overestimates of the true prevalence. When the true prevalence of vitamin A deficiency is low, the validity of the estimate depends almost entirely on specificity, and the optimal cut-point is one for which specificity is increased even at the expense of sensitivity.

These principles are illustrated in Figure 6, which shows theoretical conditions whereby combinations of values for sensitivity, specificity and true prevalence give prevalence estimates without bias due to diagnostic error (straight lines). However, the paired values of sensitivity and specificity that can be actually achieved with the combined use of three proxy markers (RBP, transthyretin and C-reactive protein) is indicated by the ROC curve. The intersect of the ROC curve and the straight lines determine the cut-point for the linear predictor that gives a prevalence estimate without bias due to diagnostic error. Interpretation of this linear predictor is relatively straightforward. For example, at a true prevalence of vitamin A deficiency of 15%, individuals for whom (−15.277 × [RBP_μmol/L_] - 7.013 × [Transthyretin_μmol/L_] + 0.367 × [C-reactive protein_mg/L_] + 24.714) > 0.496 should be classified as deficient, whereas all others can be classified as replete (in this formula, concentrations are indicated in straight brackets and expressed in units as indicated). Such classification may serve as the basis to compute the prevalence estimate.

We arbitrarily selected prevalence values of 6%, 15%, 30% and 40% as the mid-points for the ranges that indicate vitamin A deficiency as a mild, moderate or severe public health problem, and allowed the optimal cut-point for our linear predictor to vary accordingly. These cut-points enable national surveys to assess population vitamin A status at a lower cost and with more accuracy. Further research is needed to confirm whether this linear predictor yields valid results in different populations and laboratories.

It should be noted that prevalence estimates obtained using our method depend on *a priori* presumed values. This dependency is similar to clinical practice, where interpretation of test results from individual patients necessarily depends on the *a priori* presumed probability of disease. We believe, however, that our method yields more accurate results than those obtained when diagnostic inaccuracy is not taken into account.

## Conclusions

We conclude that the combination of transthyretin, RBP and C-reactive protein showed good diagnostic performance in assessing vitamin A deficiency and has great potential to eventually replace serum retinol concentration measured by HPLC as the preferred method to assess the population burden of vitamin A deficiency. Our methodology can be widely applied for other diagnostic aims.

## Additional file

Additional file 1:
**Frequency distribution of serum retinol concentration AND Validation of fluorescence method.**

## Abbreviations

AUC: area under the curve
HPLC: high-performance liquid chromatography
RBP: retinol binding protein
ROC: receiver operating characteristic
CV: coefficient of variation
