# Supplementary material for: Proxy markers of serum retinol concentration, used alone and in combination, to assess population vitamin A status in Kenyan children: a cross-sectional study
Source: BMC Med. 2015 Feb 11;13:30. doi: 10.1186/s12916-014-0256-5 (PMC4324407; doi:10.1186/s12916-014-0256-5)
Supplement: Additional file 1: — Frequency distribution of serum retinol concentration AND Validation of fluorescence method. [file 12916_2014_256_MOESM1_ESM.docx]

**Additional File**


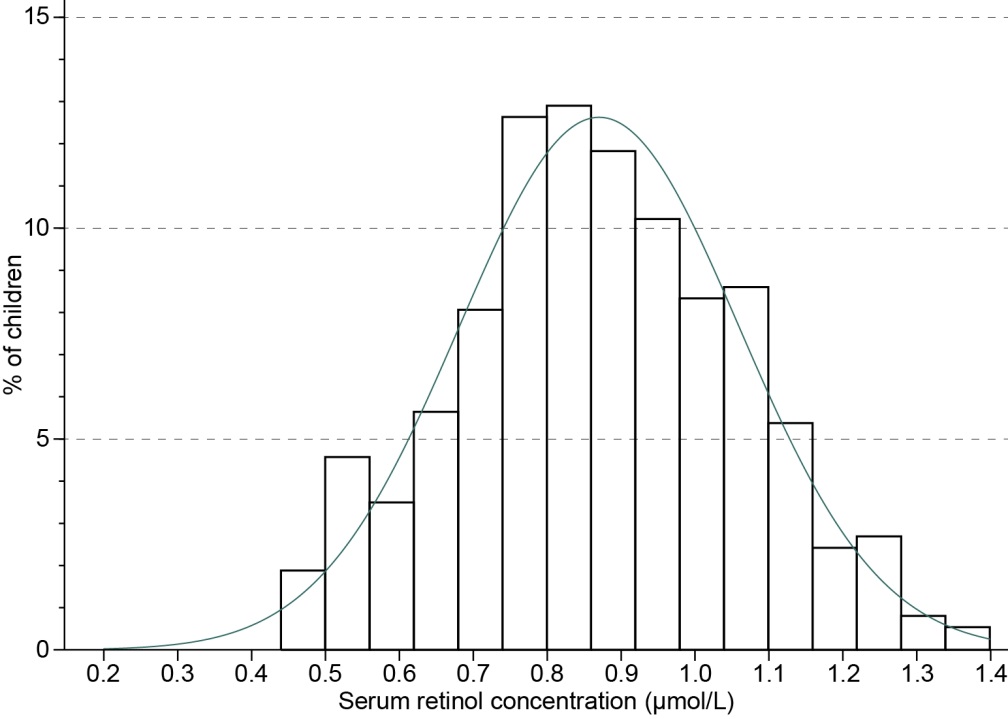


Figure 1: Frequency distribution of serum retinol concentrations as determined by HPLC in Kenyan school children

**Validation of fluorescence method to measure serum retinol concentrations**

Method: A first prototype of the fluorescence machine (iCheck) was tested in the field during sample collection in 2010. The correlation between serum retinol by HPLC and fluorescence was low (r=0.43 p<0.01), and sensitivity and specificity of fluorescence to in detecting vitamin A deficiency (defined as serum retinol<0.70 μg/L as analyzed by HPLC) were 69% and 55%. Based on our findings and recommendations, the manufacturer made adjustments to the machine and provided an improved iCheck version in 2011. This supplement shows the methods and results of a validation of the 2011 iCheck in our laboratory under controlled conditions

For this, 15 ml whole blood was obtained from 3 individuals and 3 aliquots of 5 ml were kept in the dark at 2˚- 8˚ C. Two aliquots were centrifuged at 3,000*g* for 10 minutes and serum was stored at 2–8 ˚C until analyses. For measuring fluorescence, 2×250 µL of whole blood or serum was injected into a sealed glass cuvette prefilled with a registered reagent (BioAnalyt IEX^tm^ MILA) comprising a mixture of alcohols and organic solvents. The vial was shaken vigorously for 10 seconds and left to settle for 5 minutes in dark conditions to allow separation of solvent and blood. The outer surface of the cuvette was cleaned with a tissue before it was inserted into the machine. Four readings were taken at 20 sec intervals, each time turning the vial by one-quarter. The machine automatically reports the result as the average of these four readings. For each sample, we used the average of two measurements at a one-minute interval (hence 2×4 readings). Data obtained from whole blood was adjusted for hematocrit value, which was estimated from hemoglobin levels (Hemocue 301, Ängelholm, Sweden) according to Bioanalyt iCheck guidelines. Standard control samples provided by the manufacturer were measured at the beginning and at the end of each batch of measurements and were within range.

Linearity of the relationship between serum retinol concentrations measured by fluorescence and HPLC was assessed in duplicate over a range of concentrations (100%, 70%, 50%, 35%, 20% and 0 %) in whole blood samples from two individuals diluted with phosphate-buffered saline. The prefilled cuvette used for fluorescence requires a 500 µL sample volume. To assess whether a 250 µL sample would provide similar results as a 500 µL sample we measured blood samples from two individuals as 500 µL whole blood, 500 µL serum and 250 µL serum + 250 µL phosphate-buffered saline in duplicate and compared it to HPLC concentrations. To correct for the lower serum volume in the latter we multiplied the results by two.

Retinol concentrations measured by fluorescence could potentially be influenced by storage time, temperature of the environment, and light, which are typical factors that can vary in field settings. To assess the effect of each of these factors, we first stored whole blood samples of 3 individuals in triplicate for 30 minutes, 1 hour, 7 hours and 24 hours at 4 °C in dark conditions, followed by fluorescence measurement. Secondly, we exposed 6 samples with varying concentrations to increasing temperatures (15 °C, 20 °C, 25 °C, 30 °C and 35 ˚C) in a climate room with a constant humidity of 50%-60% and artificial light. Samples and machine were allowed to adapt to the indicated temperatures for 10 minutes, starting with 15 ˚C. Lastly, we exposed 6 samples with varying concentrations to three different light conditions: 1) in the laboratory, at 4 meters removed from the window with artificial room lights on; 2) outside at mid-morning with major overcast conditions and no visible sun; and 3) in the laboratory, close to the window with artificial lights on.

Results: Linearity of retinol concentration measured by fluorescence and HPLC from two individuals was good, with high correlation and good association (Figure 2). We found no meaningful difference between results derived from whole blood samples and those from serum. At 7h and 24h of storage, concentrations were reduced by 3.5% and 12%, respectively. We found no evidence that storage for 1 hour or less influenced retinol concentrations. We found no evidence that temperature affected retinol concentrations measured by fluorescence (Figure 3). We found a clear effect of light conditions: samples measured outside and close to a window gave consistently higher retinol values than those measured inside (Figure 4).

Conclusion: Based on our results we conclude that the iCheck method provides good linearity when compared to retinol by HPLC. Light has a clear influence on the measurements and therefore the method should be used under controlled light conditions.


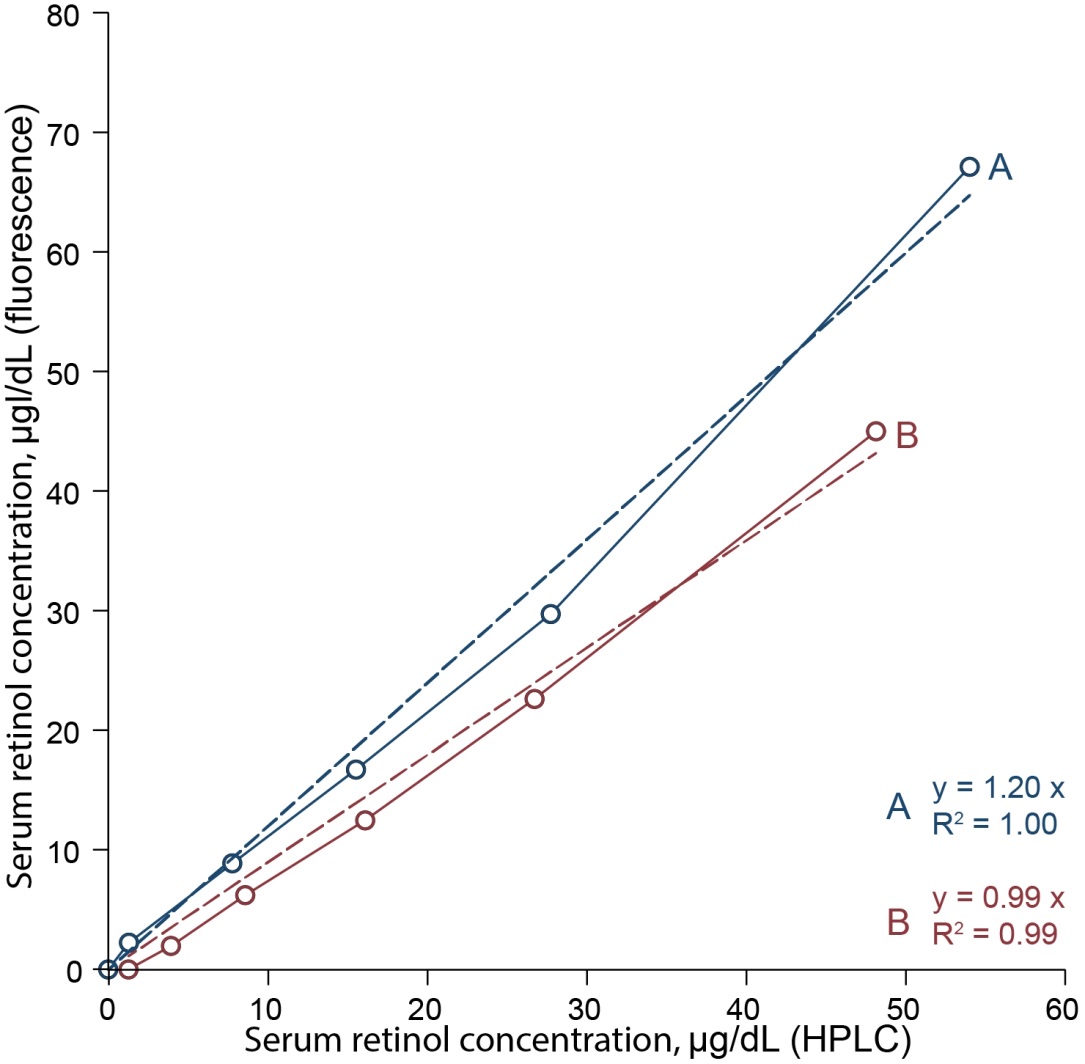


Figure 2: Linearity of retinol concentrations measured by fluorescence and HPLC in µg/dL over a dilution range of whole blood from two individuals (A and B)

Figure 3: Retinol concentrations measured by fluorescence over a temperature range of 15˚ to 30˚ in 6 different dilutions

Figure 4: Retinol concentrations measured by fluorescence during three different light conditions
